# Polyphenols and Alkaloids in Byproducts of Longan Fruits (*Dimocarpus Longan* Lour.) and Their Bioactivities

**DOI:** 10.3390/molecules24061186

**Published:** 2019-03-26

**Authors:** Ya-Yuan Tang, Xue-Mei He, Jian Sun, Chang-Bao Li, Li Li, Jin-Feng Sheng, Ming Xin, Zhi-Chun Li, Feng-Jin Zheng, Guo-Ming Liu, Jie-Min Li, Dong-Ning Ling

**Affiliations:** 1 Agro-food Science and Technology Research Institute, Guangxi Academy of Agricultural Sciences, 174 East Daxue Road, Nanning 530007, China; tangyayuan@gxaas.net (Y.-Y.T.); xuemeihegx@gxaas.net (X.-M.H.); changbaoli@gxaas.net (C.-B.L.); lili@gxaas.net (L.L.); shengjinfeng@gxaas.net (J.-F.S.); xinming@gxaas.net (M.X.); lizhichun@gxaas.net (Z.-C.L.); zhengfengjin@gxaas.net (F.-J.Z.); guoming-liu@gxaas.net (G.-M.L.); lijiemin@gxaas.net (J.-M.L.); lingdongning@gxaas.net (D.-N.L.); 2 Guangxi Key Laboratory of Fruits and Vegetables Storage-processing Technology, 174 East Daxue Road, Nanning 530007, China

**Keywords:** longan fruit byproducts, functional compositions, identification, quantification, bioactivities

## Abstract

The longan industry produces a large amount of byproducts such as pericarp and seed, resulting in environmental pollution and resource wastage. The present study was performed to systematically evaluate functional components, i.e., polyphenols (phenolics and flavonoids) and alkaloids, in longan byproducts and their bioactivities, including antioxidant activities, nitrite scavenging activities in simulated gastric fluid and anti-hyperglycemic activities in vitro. Total phenolic and total flavonoid contents in pericarp were slightly higher than those in seeds, but seeds possessed higher alkaloid content than pericarp. Four polyphenolic substances, i.e., gallic acid, ethyl gallate, corilagin and ellagic acid, were identified and quantified using high-performance liquid chromatography. Among these polyphenolic components, corilagin was the major one in both pericarp and seed. Alkaloid extract in seed showed the highest DPPH radical scavenging activity and oxygen radical absorbance capacity. Nitrite scavenging activities were improved with extract concentration and reaction time increasing. Flavonoids in seed and alkaloids in pericarp had potential to be developed as anti-hyperglycemic agents. The research result was a good reference for exploring longan byproducts into various valuable health-care products.

## 1. Introduction

Longan (*Dimocarpus longan* Lour.) belongs to the Sapindaceae family. It is an economically important subtropical and tropical plant, widely distributed in Southeast Asia countries such as China, Vietnam, Thailand and The Philippines [1]. The sweet and juicy longan fruits attract many consumers around the world and therefore market demand for them has been gradually increasing lately [2]. Longan is most often consumed as the fresh fruit, and pulp is the only edible part when consumed fresh. Other processed pulp products in the market include dried longan pulp, longan juice [3], longan jelly, longan wine [4] and canned longan in syrup. Among the products mentioned above, dried longan pulp and canned longan in syrup are the primary products.

Longan fruit is well-known owing to the match between medicine and food. The health benefits of this fruit were recorded in The Compendium of Materia Medica (Ben Cao Gang Mu in Chinese) by Li Shizhen, a famous traditional Chinese medicine expert of the Ming Dynasty, who considered longan fruit as a tonic and called it as the king of fruits. For centuries, longan pulp has been used in Traditional Chinese Medicine (TCM) as a stomachic, febrifuge, and vermifuge, and also as an antidote to poisons, etc [5,6].

Unlike longan pulp, longan pericarp and seed (accounting for approximately 20% of the weight of the whole fresh fruit) are commonly discarded as wastes, which creates many environmental problems [7]. However, longan pericarp and seed byproducts have been analyzed and found to possess high amounts of polyphenols, including proanthocyanidin A2, (–)-epicatechin, gallic acid and ellagic acid, as well as polysaccharides [8]. The powder of dried longan byproduct can be applied for treating bleeding, dampness, hernia, lymphomegaly of the neck and armpits, odour, scabies and eczema, as described in a TCM pharmacopoeia named Chinese Herbal Medicine. The National Herbal Compendium of China also records that longan byproduct powder is generally applied for stomach pain and as a styptic. The multiple medical functions of longan seeds, and especially the reduction of swelling as recorded in the TCM pharmacopoeia, imply that this TCM can be applied in cases of microbial infection, inflammation, and metabolic diseases. Evidence to this end has been revealed by current scientific methods during the past decade. Longan byproducts exhibit strong antioxidant and inflammatory activities [9,10]. Recently, several studies have further revealed that longan pericarp and seed extracts exhibit anti-cancer activity towards colorectal [11], liver [12], lung [13], cervical and breast cancer [14]. Considering their health benefits, it is necessary to effectively explore functional values of longan byproducts.

Although plenty literature has proved the potential health benefits of longan pulp, there are few reports on the bioactive substances and functional activities of longan pericarp and seed. The present study was conducted to systematically evaluate functional components in longan byproducts as well as their antioxidant activities, nitrite scavenging activities in simulated gastric fluid and anti-diabetics activities in vitro. The major functional components, i.e., polyphenolic substances, in longan byproducts were identified and quantified using high-performance liquid chromatography (HPLC) analysis. The research findings should be helpful to systematically elucidate the bioactive substances and potential functions of longan pericarp and seed, providing a scientific foundation for further exploring health-care products from longan byproducts.

## 2. Results and Discussion

### 2.1. Polyphenols in Different Parts of Longan Fruit

#### 2.1.1. Total Phenolic Content (TPC) and Total Flavonoid Content (TFC)

Polyphenolic substances are secondary metabolites of plants with various functions such as protection against pathogens and predators, mechanical support, attraction of pollinating animals, and prevention of ultraviolet radiation [15]. Amongst polyphenolic substances, flavonoids are the largest subclass. Their chemical structures contain two or more aromatic rings and each bears at least one aromatic hydroxyl connecting with a carbon bridge [16]. In this study, TPC and TFC were significantly different (*P* < 0.05) among extracts from longan pericarp, seed and pulp. TPC and TFC in longan pericarp were slightly higher than those in longan seed (Table 1), which was also reported by other researchers [17]. Longan pericarp and seed possessed significantly higher (*P* < 0.05) TPC and TFC than longan pulp (Table 1), and therefore phenolics and flavonoids from longan pericarp and seed were principally chosen for the following bioactivity analysis.

TPC and TFC of longan fruits varied under different environments and regions. In the Chinese market, there are many popular longan cultivars such as cv. Shixia and cv. Linglong from Guangxi Province, cv. Chuliang from Guangdong Province, cv. Gushan No.2 from Fujian Province, etc. A huge difference of phenolic contents existed in 18 longan cultivars. Among these cultivars, cv. Hualu Guangyan showed the maximum phenolic content (0.75 mg GAE/g), and cv. Jiluanyan possessed the minimum phenolic content (0.013 mg GAE/g) [18]. They found that TPC in pulp of cv. Shixia was around 0.63 mg GAE/g, but TPC in pulp of cv. Shixia was only about 0.08 mg GAE/g in this research.

#### 2.1.2. Identification and Quantification of Individual Polyphenolic Compounds

**Figure 1:**
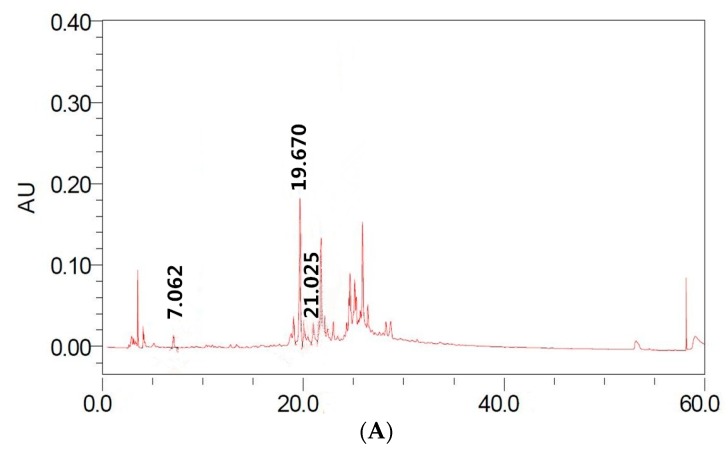

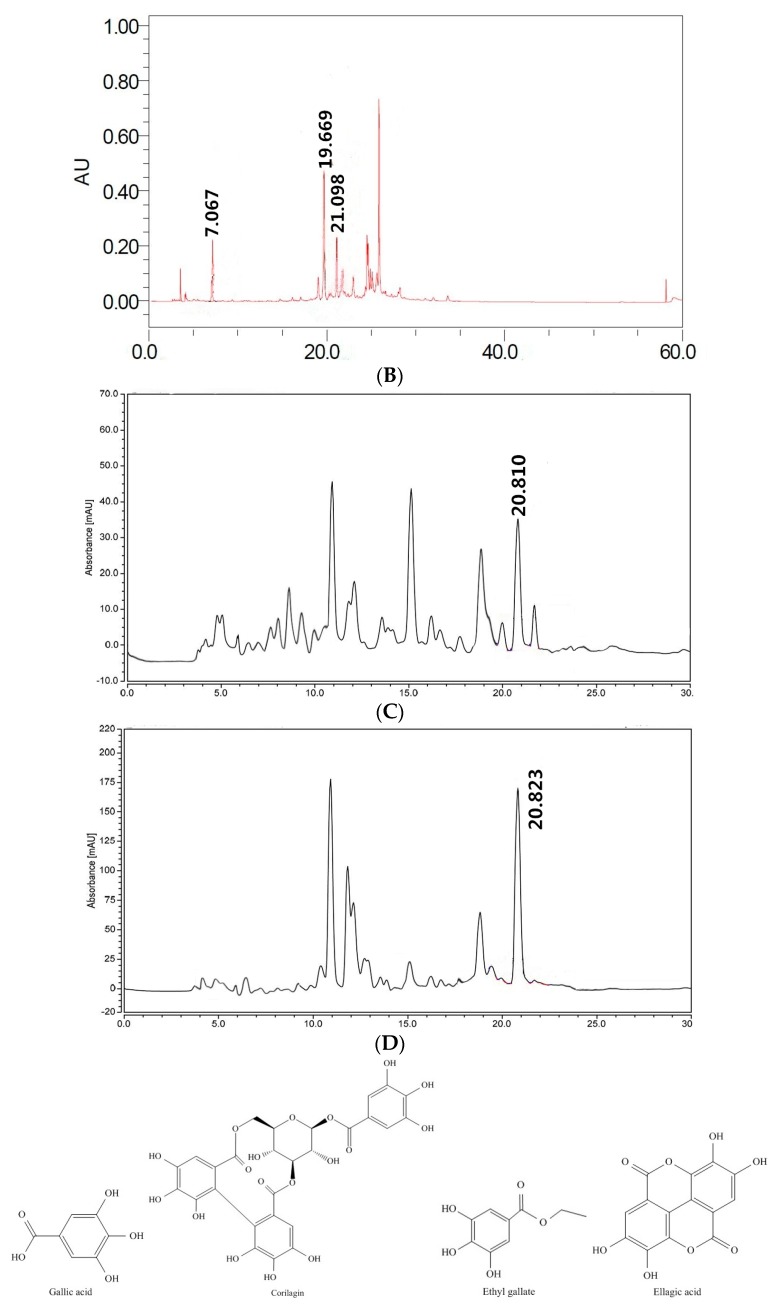
HPLC chromatograms and chemical structures of gallic acid, corilagin and ethyl gallate in longan pericarp extracts (**A**) and longan seed extracts (**B**) detecting at 270 nm; HPLC chromatograms and chemical structure of ellagic acid in longan pericarp extracts (**C**) and longan seed extracts (**D**) detecting at 260 nm.

TPC determination based on color reaction may sometimes overestimate polyphenolic content, because other non-phenolic substances also exhibit positive reaction [19]. Hence, polyphenol quantification was further performed by HPLC analysis. In this research, four polyphenols, i.e., gallic acid, ethyl gallate, corilagin and ellagic acid, were identified in longan pericarp and seed extracts. The retention time (RT) of gallic acid, corilagin and ellagic acid were around 7.06 min, 19.6 min and 21.0 min, respectively (Figure 1). The RT of ellagic acid was around 20.8 min (Figure 1). Longan pericarp and seed extracts showed significant differences (*P* < 0.05) in their individual polyphenolic contents (Table 2). The contents of four bioactive compounds (gallic acid, ethyl gallate, corilagin and ellagic acid) in longan seed were 2–5 times higher than those in longan pericarp. For instance, using 70% ethanol as extraction solution, ellagic acid content in longan seed (about 0.18 mg/g) was nearly five times higher than that in longan pericarp (about 0.83 mg/g) (Table 2). Hence, longan seed could be a good source for extracting gallic acid, ethyl gallate, corilagin and ellagic acid. In addition, different extraction methods obviously affect polyphenolic content. Various solvents such as water, ethanol, acetone and methanol are often used for extracting polyphenolic substances. Ethanol is a widely used “green” solvent among them. Therefore, this study focused on extraction efficiency of 50% and 70% ethanol to four polyphenolic compounds in longan byproducts. From Table 2, 50% ethanol extracts of pericarp and seed contained lower contents of four polyphenols than 70% ethanol extracts, which was also mentioned by other researchers [1]. Corilagin was the major polyphenolic compound in both pericarp (2.15 ± 0.08 mg/g) and seed (5.53 ± 0.05 mg/g). However, gallic acid content was the lowest (only about 0.08 mg/g and 0.49 mg/g) in both tissues. Corilagin possesses several important pharmacological activities such as fighting against fungus *Candida glabrata* strains [20], potently inhibiting HIV-1 replication in HeLa CD4^+^ cells [21], and inhibiting the release of tumor necrosis factor-R (TNF-R) [22]. It is also related to the lowering of blood pressure and the prevention of cardiovascular disease.

### 2.2. Total Alkaloid Content (TAC) in Different Parts of Longan Fruit

Alkaloids are another important group of secondary metabolites in plants, which are amino acid-derived basic nitrogen-containing organic components [23]. For centuries, alkaloids in plant are frequently utilized as remedies, central nervous system stimulants, potions or poisons. At present, alkaloids are well-known as neuroprotective agent, anti-cancer agent, cardioprotective agent, anti-diabetic agent, immune stimulant, anti-inflammatory agent, anti-viral drug, etc [24].

Most studies focus on bioactive alkaloids in medicinal plants, such as *Folium mori* and *Sophora flavescens*. However, there are only a few reports on alkaloids in fruits, for example synephrine in citrus [25]. In fact, fruits contain various alkaloids which also possess special bioactivities. Just like citrus, longan is an excellent fruit with the homology of medicine and food. Unfortunately, unlike polyphenols, few researches related to alkaloids in longan fruit have been performed up to date. The extraction method of total alkaloids was discussed and modified, and under the best parameters, the extraction rate could be around 2.33% [26]. In the present study, the extracts from different parts (pericarp, seed and pulp) of longan fruit showed significant differences (*P* < 0.05) in total alkaloid content. From Table 1, longan seed possessed higher TAC value (7.40 ± 1.04 mg HE/g) than pericarp (6.44 ± 0.23 mg HE/g). Moreover, TAC values in longan pericarp and seed were significantly higher (*P* < 0.05) than those in longan pulp (1.67 ± 0.13 mg HE/g). Therefore, alkaloids from longan pericarp and seed were also analyzed in following functional experiments.

### 2.3. Functional Activities of Polyphenolic and Alkaloid Extracts in Longan Byproducts

#### 2.3.1. Antioxidant Activities

**Figure 2:**
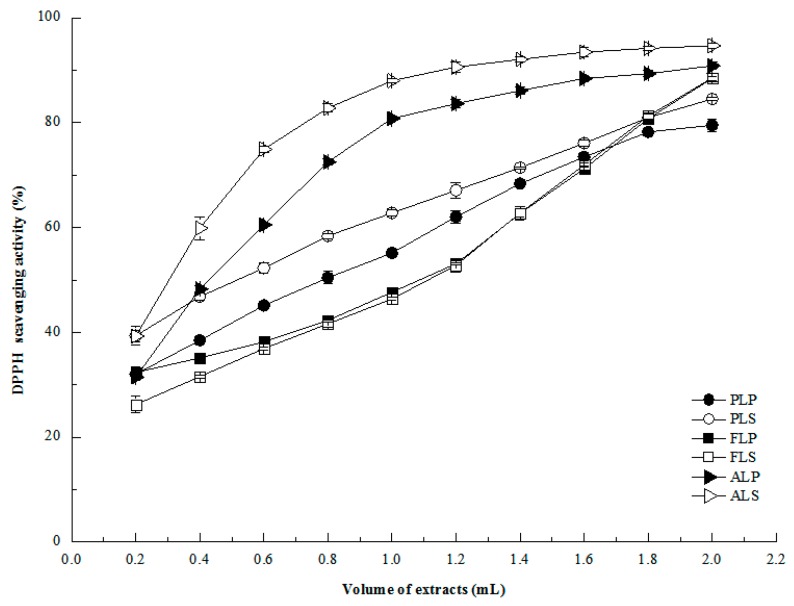
DPPH radical scavenging activities of longan byproducts. PLP: phenolic in longan pericarp, PLS: phenolic in longan seed, FLP: flavonoid in longan pericarp, FLS: flavonoid in longan seed, ALP: alkaloid in longan pericarp, and ALS: alkaloid in longan seed. Phenolics in longan pericarp and seed were diluted 2 times, flavonoids in longan pericarp and seed were diluted five times, and alkanoids in longan pericarp and seed were diluted 100 times.

Polyphenols (e.g., phenolics and flavonoids) play a crucial role in reducing chronic metabolic syndrome (e.g., diabetes) and cardiovascular disease, and also act as regulators of cell signal and cellular process in cancer prevention and treatment [27]. The antioxidant capacity (scavenging ROS/RNS or preventing their formation) of polyphenols is the most well-known and acceptable mechanism related to health [16]. Alkaloids have also been proven as antioxidants and radical scavengers in some traditional Chinese medicines, teas and fruits. The present study mainly analyzed antioxidant activities of polyphenolic and alkaloid extracts from longan byproducts using DPPH and ORAC methods. In general, from IC_50_ values (Table 3), longan seed alkaloids (22.90 ± 1.20 μg extract/g) possessed the maximum DPPH radical scavenging activity among all extracts, followed by pericarp flavonoids (451.96 ± 3.62 μg extract/g) and seed flavonoids (427.66 ± 3.01 μg extract/g). This result indicated that longan seed alkaloids possessed the best antioxidant activity with the lowest value of half maximal inhibitory effect among all extracts. As for phenolic extracts, seed phenolics (1007.89 ± 23.22 μg extract/g) exhibited a significantly (*P* < 0.05) stronger antioxidant activity than pericarp phenolics (1827.11 ± 16.64 μg extract/g). From Figure 2, it was also found that polyphenols and alkaloids in longan pericarp and seed showed a dose-dependent scavenging activity against DPPH radicals.

**Figure 3:**
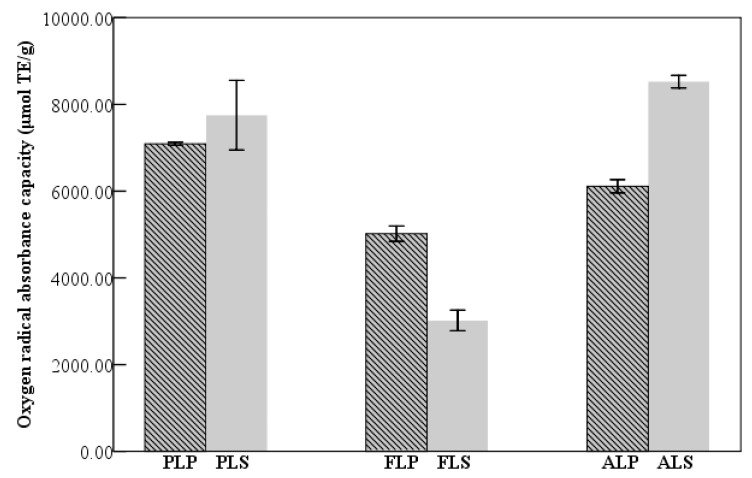
Oxygen radical absorbance capacities of longan byproducts. PLP: phenolic in longan pericarp, PLS: phenolic in longan seed, FLP: flavonoid in longan pericarp, FLS: flavonoid in longan seed, ALP: alkaloid in longan pericarp, ALS: alkaloid in longan seed, and TE: Trolox equivalent.

The specificity and sensitivity of DPPH assay did not completely confirm antioxidant activities of these extracts. Hence, ORAC assay was further conducted to provide reliable assessment on antioxidant properties of longan byproducts. Trolox (3.125 μM to 100 μM) was used as a standard for this assay. From ORAC value (Figure 3), longan seed alkaloids (8524.69 ± 206.06 μmol TE/g) showed the maximum antioxidant capacity among all extracts, followed by seed phenolics (7750.76 ± 1135.56 μmol TE/g), pericarp phenolics (6868.22 ± 386.22 μmol TE/g) and pericarp alkaloids (6112.69 ± 217.59 μmol TE/g). However, flavonoids in longan byproducts were relatively lower than other extracts. Some literatures have showed different relationships between antioxidant activities and contents of polyphenols and alkaloids. A negative correlation between antioxidant capacity and total phenolic content was found in strawberry, a positive relation was reported in peach, but no correlation was observed in apricot [28]. In the present research, a positive correlation between antioxidant capacity and content was only found in longan alkaloid extracts, and yet a negative relationship was exhibited in longan polyphenolic extracts (Table 1 and Table 2 and Figure 2), indicating that diverse phytochemical compounds in longan byproducts contributed to the relationship variation.

Moreover, according to previous literature, longan fruits have plenty of compounds with “antioxidant capacity”. In fact, the term “antioxidant capacity” has many different meanings in different contexts, such as free radical scavenging capacity, oxidation inhibition capacity, or disease prevention capacity [16]. Therefore, it is important to specify “antioxidant capacity”, correlating with particular experiments. In other words, there is the lack of correlation between different methods for the same antioxidants [29]. Hence, there should be no direct comparison between DPPH and ORAC assessment.

#### 2.3.2. Nitrite Scavenging Activities

Nitrite or nitrate in food is closely related to human health. Smoked products and preserved meats (e.g., pork, fish, sausage, etc.) are important sources of nitrite or nitrate [30]. In the stomach under acidic conditions, nitrite reacts with secondary amines to easily form N-nitrosamines (NAs) [31]. NAs, known as one of the strongest chemical carcinogens, are related to an increased risk of nasopharyngeal, esophageal, gastric, liver, colon and bladder cancers [32]. Human can be exposed to carcinogenic NAs from endogenous and exogenous sources. There are many modulators of endogenous N-nitroso compound (NOC) synthesis. Chemical inhibitors such as ascorbic acid, tocopherol and phenolic compounds, can regulate the endogenous formation of NOCs [33,34].

Longan extracts contain functional compounds such as polyphenols and alkaloids, which possess potential as nitrite scavengers to protect against tumor formation [35]. Unfortunately, there is still no research on longan extracts as nitrite scavengers under simulated gastric fluid conditions. Experiments simulating gastric fluid condition, digestion time and digestion process can directly illustrate any potential changes of longan extracts in the human body. Compared with animal studies, these experiments are easier to control. Therefore, we chose to determine the effects of longan extracts on nitrite scavenging activities.

Generally, there are two ways, i.e., enzymatic degradation and non-enzymatic degradation, for nitrite removal. The enzymatic degradation pathway relies mainly on xanthine oxidoreductase, nitrite reductase and nitrite synthase to make reduce nitrite to NO [36]. The non-enzymatic degradation pathway is based on a number of strong reducing substances (e.g., vitamin C and flavonoids) [37]. In this pathway, nitrite is reduced to NO, N_2_O, H_2_N_2_O_2_, N_2_ and other substances [38], and is ultimately degraded. In this study, the nitrite scavenging activities of longan extracts might be related to their strong reducing substances such as phenolics and alkaloids. Table 4, Table 5 and Table 6 present the nitrite scavenging activities of longan extracts for different volumes and reaction times in simulated gastric fluid. Longan pericarp and seed extracts showed a significant (*P* < 0.05) difference in nitrite scavenging activities. The scavenging activity was improved with the increase of extract quantity. There was a positive correlation between reaction time and scavenging rate. Scavenging rates increased significantly with reaction time in the 10–60 min range. The nitrite scavenging activities of all extracts reached peaks (up to 90%) after treating for 60 min. Under the same extract volume and reaction time, the nitrite scavenging activity of longan seed phenolics was a little stronger than that of pericarp phenolics (Table 4), whereas an opposite result was demonstrated in pericarp and seed flavonoid extracts (Table 5). Nitrite scavenging ability of pericarp alkaloids was much stronger than that of seed alkaloids (Table 6). Some researchers have reported a positive relationship between longan polyphenol concentration and nitrite scavenging ability [39], however, in the current study, except for longan flavonoid extracts, nitrite scavenging abilities of phenolic and alkaloid extracts were not dependent on TPC and TAC (Table 1). This might be due to each group of components contained many compounds with different chemical structures, which can affect their abilities in nitrite removal. Further research combining with NMR could be done for the hypothesis.

#### 2.3.3. Anti-Hyperglycemic Activities

**Figure 4:**
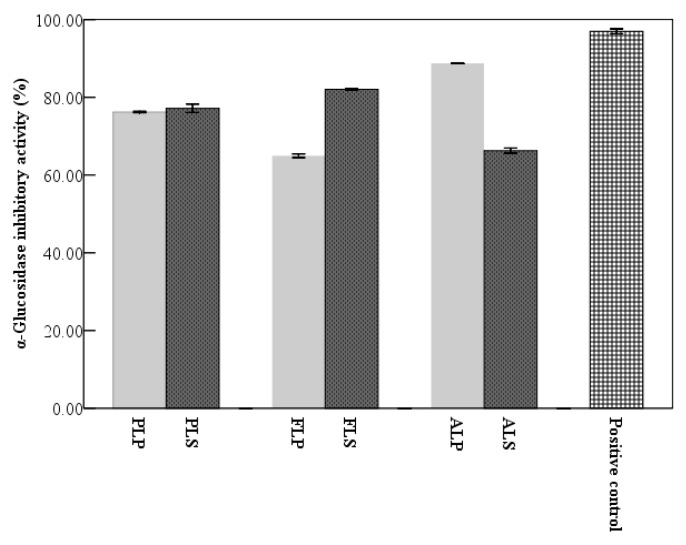
α-Glucosidase inhibitory activities of longan byproducts. PLP: phenolic in longan pericarp, PLS: phenolic in longan seed, FLP: flavonoid in longan pericarp, FLS: flavonoid in longan seed, ALP: alkaloid in longan pericarp, and ALS: alkaloid in longan seed. Each extract was diluted 10 times.

Diabetes mellitus (DM) is a well-known chronic metabolic disorder. Its morbidity and mortality rates, accounting for 9% of deaths, are growing in recent decades. This has prompted efforts to explore new therapeutic agents to block the progress of DM [40]. A therapeutic approach is to retard glucose absorption through inhibiting carbohydrate-hydrolyzing enzymes, e.g., α-glucosidase, in the digestive organs [41]. Since the 1980s, only a few α-glucosidase inhibitors have become commercially available such as acarbose, miglitol and voglibose. However, all of them are associated with serious gastrointestinal side effects (nausea, vomiting, bloating, diarrhea, loss of appetite, etc.) [42]. These synthetic α-glucosidase inhibitors are expensive as well. It is necessary to effectively explore natural α-glucosidase inhibitors as alternatives for patients to alleviate their pain and suffering. Some researchers have investigated the anti-hyperglycemic activities of longan pulp, but only a few studies have been performed to identify the anti-hyperglycemic effects of longan byproducts to date. Longan pericarp extracts improve mouse glucose tolerance by oral gavage owing to the increased gene expression in insulin signalling pathway, and therefore they may act as a potential anti-hyperglycemic agent in diabetes care [43]. In the present study, phenolics, flavonoids and alkaloids extracts from longan pericarp and seed could inhibit α-glucosidase to different degrees (Figure 4). According to Table 1 and Figure 4, there was no direct relationship between longan extract concentrations and α-glucosidase inhibitory abilities. In other word, α-glucosidase inhibitory capacity was not improved by an increasing concentration of bioactive components in longan byproducts. Comparing with longan seed flavonoid (193.37 ± 0.55 mg RE/L, α-glucosidase inhibitory capability 81.63 ± 0.81%), pericarp flavonoid (207.97 ± 3.84 mg RE/L, α-glucosidase inhibitory capability 64.96 ± 0.65%) was weaker in inhibiting α-glucosidase. Phenolics in longan pericarp (75.75 ± 0.84%) showed lower α-glucosidase inhibitory activity than those in seed (77.23 ± 1.09%), yet the opposite results were found in alkaloids from longan byproducts. Longan seed alkaloids exhibited a significant lower (P < 0.05) α-glucosidase inhibitory ability (739.64 ± 20.15 mg HG/L, α-glucosidase inhibitory capability 66.06 ± 0.77%) than pericarp alkaloids (644.15 ± 13.22 mg HG/L, α-glucosidase inhibitory capability 88.72 ± 0.12%). The above results indicate that α-glucosidase inhibitory activity might be directly related to the chemical structures of longan byproduct extract components. From Figure 4, it was also observed that the α-glucosidase inhibitory activity of acarbose (positive control) was more than 95%. Among all extracts of longan byproducts, seed flavonoids and pericarp alkaloids exhibited closer inhibitory activities to positive control. Hence, longan seed flavonoids and pericarp alkaloids had potential to be developed as an anti-hyperglycemic agent. Their activities related to insulin signaling pathways would be an interesting direction for future study.

## 3. Materials and Methods

### 3.1. Chemicals and Reagents

Fluorescein disodium salt (BS), *p*-nitrophenyl glycopyranoside (PNPG, S10137), 6-hydroxy-2,5,7,8-tetramethylchroman-2-carboxylic acid (Trolox), 2,2′-azobis(2-amidinopropane) dihydrochloride (AAPH), 4-hydroxypiperidine, α-glucosidase (S10049, 100,000 U/g) as well as HPLC-grade rutin, gallic acid, ethyl gallate, corilagin and ellagic acid were purchased from Shanghai Yuanye Biological Technology Co. (Shanghai, China). Pepsin (P7000, 800-2500 U/mg), 2-diphenyl-1-picrylhydrazyl (DPPH) as well as HPLC-grade acetonitrile and methanol were obtained from Sigma-Aldrich Co. (St. Louis, MO, USA). Anhydrous sulfanilic acid, *N*-(1-naphthyl) ethylenediamine dihydrochloride, phosphoric acid and sodium hydroxide were obtained from Tianjin Damao Chemical Reagent Co. (Tianjin, China). Absolute ethanol was supplied by Chengdu Kelong Chemical Reagent Co. (Chengdu, Sichuan, China). Disodium hydrogen phosphate dodecahydrate, gallic acid monohydrate and aluminum nitrate nonahydrate were purchased from Tianjin Kermel Chemical Reagent Co. (Tianjin, China). Hydrochloric acid (≥36%) was obtained from Lianjiang Ailian Chemical Reagent Co. (Lianjiang, Guangdong, China). Folin-Ciocalteu reagent was supplied from Beijing Solarbio Science & Technology Co. (Beijing, China). Reinecke salt was purchased from Sinopharm Chemical Reagent Co. (Beijing, China). Other chemical reagents were offered by Tianjin Bodi Chemical Reagent Co. (Tianjin, China). All chemicals were of analytical grade unless they were specially mentioned.

### 3.2. Preparation of Longan Fruit Samples

Longan fruits (cv. Shixia, a common cultivar in China) were obtained from Haijixing Fruit & Vegetable Market in Nanning (Guangxi Province, China) in August 2017. The fresh longan fruits were immediately separated into pulp, pericarp and seed once they were transported to the Guangxi Key Laboratory of Fruits and Vegetables Storage-processing Technology. After drying for 12 h at 60 °C, all samples were crushed into powder using a FW 177 high rotated speed disintegrator (Taisite Instrument Co., Tianjin, China). The samples were then stored in a cool and dark shelter for further studies.

### 3.3. Determination of Bioactive Substances in Different Parts of Longan Fruit

#### 3.3.1. Determination of Total Phenolic Content (TPC)

Accurately weighed longan pericarp and seed powder (1 g) as well as longan pulp (control) was extracted for 1.5 h in a 100-mL Erlenmeyer flask placed in a 70 °C water bath using 50% ethanol (40 mL). Next the extract was centrifuged for 15 min at 3000× *g*, and the supernatant was collected. The residue was re-extracted with 50% ethanol (40 mL). The extracts were combined, filtered and diluted to 100 mL using 50% ethanol in a volumetric flask. All extracts were transferred to 50-mL capped centrifuge tubes and stored at 4 °C in the dark. TPC was evaluated by a modified Folin-Ciocalteu assay method using gallic acid (GA) as an external standard [40]. At room temperature, the extract (1 mL) was added with distilled water (5 mL) and Folin-Ciocalteu reagent (1 mL). Then, the mixture was left standing for 5 min and 20% Na_2_CO_3_ (3 mL) was added. The mixture was vortexed and incubated for 1 h in the dark. The absorbance was measured on a Genesys 10S UV-Visible spectrophotometer (Thermo Fisher Scientific Inc., Waltham, MA, USA) at 765 nm against distilled water (as blank). TPC was calculated using a calibration curve of GA with concentration ranged from 0.001 to 0.005 mg/mL (r^2^ > 0.99). Results were expressed as milligrams of GA equivalents per gram of longan dried sample (mg of GAE/g longan sample).

#### 3.3.2. Determination of Total Flavonoid Content (TFC)

Longan pericarp and seed powder (precisely 1 g) as well as longan pulp (control) were extracted for 1 h in a 100-mL Erlenmeyer flask in a 70 °C water bath using 70% ethanol (40 mL). After the extract was centrifuged for 15 min at 3000× *g*, the supernatant was collected. The residue was re-extracted with 70% ethanol (40 mL). The extracts were combined, filtered and diluted to 100 mL using 70% ethanol in a volumetric flask. All extracts were transferred to 50-mL capped centrifuge tubes and stored at 4 °C in the dark. TFC was determined by a modified colorimetric method using rutin as a standard [44]. The extract (1 mL) was mixed with 0.7 mL of 5% NaNO_2_ solution before stood for 5 min, and then 0.7 mL of 10% Al(NO_3_)_3_ solution was added. After 6 min, 5 mL of 1 M NaOH was added to the mixture and finally diluted to 25 mL using 70% ethanol in a volumetric flask. The absorbance was instantly measured at 510 nm against distilled water (as blank). TFC was calculated using a calibration curve of rutin with concentration ranged from 0.008 to 0.048 mg/mL (r^2^ > 0.99). Results were expressed as milligrams of rutin equivalents per gram of longan dried sample (mg of RE/g longan sample).

#### 3.3.3. Determination of Total Alkaloid Content (TAC)

Two grams of longan pericarp and seed powder as well as longan pulp (control) were extracted in a 100-mL Erlenmeyer flask at ambient temperature for 3 h using 25% ethanol/HCl (40 mL, 0.05 M). After the extract was centrifuged for 15 min at 3000× *g*, the supernatant was collected. The residue was re-extracted using the same procedure. The extracts were combined, filtered and diluted to 100 mL using 25% ethanol/HCl (0.05 M) in a volumetric flask. All extracts were transferred to 50-mL capped centrifuge tubes and stored at 4 °C in the dark. TAC was analyzed using a previous published method with slight modification [45]. The extract (5 mL) was mixed with 5 mL of 0.1 M HCl and 3 mL of 2% freshly prepared Reinecke salt. The mixture in a 25-mL volumetric flask was vortexed and incubated for 30 min in ice-water bath. Then, the mixture was centrifuged for 10 min at 3000× *g* and the supernatant was removed. The precipitate was dissolved with acetone and diluted to 5 mL. The absorbance was determined at 525 nm against distilled water (as blank). 4-Hydroxypiperidine was used as external standard. TAC was calculated using the calibration curve of 4-hydroxypiperidine with concentration ranged from 1 to 5 mg/mL (r^2^ > 0.99). Results were expressed as milligrams of 4-hydroxypiperidine equivalents per gram of longan dried sample (mg of HE/g longan sample).

### 3.4. Identification of Major Polyphenolic Compounds in Longan Byproducts

Polyphenolic compounds from longan byproducts were extracted according to the method of Sun et al. [29] with some modification. Longan pericarp or seed powder (1 g) was mixed with 50% ethanol or 70% ethanol (40 mL or each) in a 100-mL Erlenmeyer flask. The mixture was vigorously shaken for 1.5 h in water bath at 70 °C. After the sample was centrifuged for 10 min at 3000× *g*, the supernatant was transferred into a 100-mL volumetric flask. The residue was re-extracted with ethanol using the same procedure. All extracts were combined and diluted to 100 mL using 50% ethanol or 70% ethanol. Each extract was concentrated to 25 mL in a rotary evaporator, and filtered through a 0.45 μm PVDF membrane prior to HPLC analysis. In addition to two different extraction solvents, polyphenolic compounds were extracted with the same extraction procedure.

Standard solutions of gallic acid, ethyl gallate and corilagin were prepared by dissolving these standards into 0.5, 5, 10, 20, 50, 80, 100 and 200 μg/mL, and ellagic acid was dissolved into 1, 5, 10, 20 and 100 μg/mL. The calibration curves of authentic standards were plotted using peak area against concentration by duplicate injections of diluted working solutions. Linear regression was used to calculate the parameters of Y = *a*X + *b*, where X was the concentration of each polyphenolic compound and Y was peak area, and *a* was slope factor. Gallic acid, ethyl gallate and corilagin in longan byproducts were identified using a Waters e2695 HPLC system (Waters, Milford, MA, USA.) equipped with a Waters 2998 Photodiode Array Detector (Waters) and a ZORBAX SB-Aq C18 column (250 × 4.6 mm, Agilent Technologies Inc., Santa Clara, CA, USA). A total of 10 μL of sample solution prepared as described above was injected into the HPLC system. The mobile phase consisted of acetonitrile (A) and 0.1% phosphoric acid solution (B). Flow rate was 1 mL/min. The gradient elution was conducted as follows: 0 min, 5% A; 10 min, 11% A; 20 min, 20% A; 21 min, 25% A; 50 min, 40% A; 50.1 min, 90% A; 55 min, 90% A and 55.1–60 min, 5% A. Column temperature was set at 30 °C. The detection was carried out at 270 nm. Furthermore, ellagic acid in longan byproducts was identified using an Ultimate 3000 HPLC (Thermo Fisher Scientific, Waltham, MA, USA) with a Phenomenex C18 column (250 × 4.6 mm, Phenomenex Inc., Torrance, CA, USA). A total of 5 μL of sample solution prepared as described above was injected into the HPLC system. A gradient elution with the mobile phase consisting of methanol (A) and 0.1% phosphoric acid solution (B) at a flow rate of 0.6 mL/min was conducted. The gradient elution was as follows: 0 min, 30% A; 15 min, 60% A; 20–25 min, 85% A and 25.1–30 min, 30% A. The detector was carried out at 260 nm. Column temperature was set at 25 °C.

### 3.5. Bioactivities of Longan Byproducts

Phenolics, flavonoids and alkaloids in longan byproducts were extracted according to above-mentioned methods and used for determining their bioactivities.

#### 3.5.1. Antioxidant Activities

##### DPPH Free Radical Scavenging Activity

DPPH free radical scavenging capacity was determined according to the modified method [39]. Different volumes (0.2–2 mL) of longan extracts and absolute ethanol (as blank) were diluted to 2 mL individually with distilled water. At room temperature, 2 mL of 6.5 × 10^−5^ mol/L DPPH reagent was added and vortexed before stood for 30 min in the dark. Absolute ethanol (2 mL) was used as a negative control in which 2 mL of DPPH reagent was added. The absorbance was read at 517 nm. The percentage of DPPH discoloration by sample was calculated according to the following equation: the percentage of discoloration = [1 − (A_sample_ − A_blank_)/A_negative control_] × 100.

##### Oxygen Radical Absorbance Capacity (ORAC)

ORAC assay was performed using the modified method of Ma et al. [46]. Synergy^TM^ HTX Multifunctional Microplate Reader (BioTek, Winooski, VT, USA) was used to monitor this reaction. The incubation temperature was set at 37 °C. The fluorescence filters with excitation wavelength of 485 nm and emission wavelength of 528 nm were used. AAPH was used as a peroxyl generator and Trolox was used as a standard. Longan extracts, blank and Trolox calibration solutions were loaded to clear polystyrene 96-well microplates (Costar 3599, Corning Inc., New York, NY, USA). Kinetic reading was recorded for 61 cycles with 2 min per cycle setting. Longan extracts and Trolox were diluted to the suitable concentration range using 75 mM phosphate buffer saline (pH 7.4). After loading 20 μL of extracts, standard and phosphate buffer saline (as blank) as well as 160 μL of fluorescein disodium salt solution into appointed wells according to the layout. The microplate (sealed with film) was incubated for 30 min in a plate reader, and then 20 μL of AAPH (153 mM) was added to initiate oxidation reaction. The kinetics of fluorescence changes were immediately recorded by software Gen5TM (BioTek, Winooski, VT, USA). The final ORAC values were calculated using a linear equation between Trolox or extract concentration and net area under fluorescence decay curve. The area under a curve (AUC) was calculated as AUC = 0.5 [2 (f_0_/f_0_ + f_1_/f_0_ + …… + f_n-1_/f_0_ + f_n_/f_0_) − f_0_/f_0_ − f_n_/f_0_] × ΔT, where ΔT was time interval between two kinetic reading, f_0_ was fluorescence reading at reaction initiation and f_n_ was last measurement. Net AUC was obtained by following equation: net AUC = AUC_Trolox or sample_ − AUC_blank_). ORAC value was calculated and expressed as micromoles of Trolox equivalent per gram sample (μmol of TE/g) using the calibration curve of Trolox. The linear range of calibration curve was 6.25 to 100 μM (r^2^ > 0.99). 

#### 3.5.2. Nitrite Scavenging Activities in Simulated Gastric Fluid

Nitrite scavenging activities under simulated gastric juice conditions were measured using a modified colorimetric method [47]. One liter of simulated gastric fluid was prepared from 2 g of sodium chloride, 3.2 g of pepsin and 7 mL of concentrated hydrochloric acid. Different volumes (0.2 to 1 mL) of longan extracts were diluted to 2 mL individually with distilled water in a 25-mL volumetric flask. Then, 2 mL of 5 μg/mL NaNO_2_ was added to the diluted extract sample and incubated in water bath at 37 °C. After 10, 30 and 60 min, 2 mL of 0.4% sulfanilic acid was immediately added, and stood for 5 min. Then, 1 mL of 0.2% N-(1-naphthyl)ethylenediamine dihydrochloride was added to the mixture. After 15 min, the mixture was diluted to 25 mL with distilled water and mixed thoroughly using vortex. The color intensity at 538 nm was measured, and the activity was expressed as nitrite scavenging activity (%) = (A_blank_ + A_negative control_ − A_sample_)/A_blank_ × 100, where A_sample_ was the absorbance of sample with NaNO_2_, A_negative control_ was the absorbance of sample without NaNO_2_, and A_blank_ was the absorbance of distilled water.

#### 3.5.3. α-Glucosidase Inhibitory Assay In Vitro

α-Glucosidase inhibitory activity was analyzed using PNPG as a substrate according to the modified method of Kang et al. [48]. A total of 100 μL of longan extract or 8 mg/L acarbose (as positive control) or distilled water (as black) was mixed with 50 μL of 8 U/L α-glucosidase and 2 mL of 0.05 M phosphate buffer (pH 6.8). After incubating for 10 min at 37 °C, 50 μL of 8 mM PNPG solution was added. The reaction was terminated by adding 2 mL of 0.1 M Na_2_CO_3_ solution after incubated for 10 min at 37 °C. Enzymatic activity was quantified by measuring the *p*-nitrophenol released from PNP-glycoside at 405 nm against a blank. Enzymatic inhibition data were calculated as the percentage of inhibition = [1 − (A_sample or acarbose_ − A_sample blank_)/A_blank_] × 100, where A_blank_ was the absorbance of blank, A_sample or acarbose_ was the absorbance of sample or positive control, and A_sample blank_ was the absorbance of sample without α-glucosidase and PNPG.

### 3.6. Statistical Analysis

Except HPLC analysis, all assays were conducted in triplicate. Data were expressed as mean ± standard deviation (SD). The statistically analysis was performed using SPSS 19.0 software (IBM Co., Chicago, IL, USA) with significance level set at *P* < 0.05.

## 4. Conclusions

The current findings can assist the public to systematically understand the bioactive substances and potential functions in longan pericarp and seed. They also serve as a good reference for future researchers or the longan industry to develop these byproducts into different valuable health-care products. Longan byproducts are rich in alkaloids with functional activities, which are presented for the first time. Polyphenols and alkaloids from longan pericarp and seed extracts have been proven as candidates responsible for bioactivity such as antioxidants, nitrite-scavenging and α-glycosidase inhibition activities. In our next report, alkaloid compounds in longan byproducts will be identified. The functional activities of longan polyphenols and alkaloids will also be further ascertained in future using animal models.

## Figures and Tables

**Table 1:** Total phenolic, flavonoid and alkaloid contents in longan fruits.

Sample	TPC (mg GAE/g)	TFC (mg RE/g)	TAC (mg HE/g)
Pulp (control)	0.08 ± 0.00 ^c^	0.10 ± 0.01 ^c^	1.67 ± 0.13 ^c^
Pericarp	4.59 ± 0.25 ^a^	2.08 ± 0.04 ^a^	6.44 ± 0.23 ^b^
Seed	3.77 ± 0.08 ^b^	1.94 ± 0.01 ^b^	7.40 ± 1.04 ^a^

Values marked by different letters within a same column were significantly different (*P* < 0.05).

**Table 2:** Quantification of polyphenolic compounds in longan byproducts by HPLC analysis.

Phenolic Compounds	Phenolic Content in Longan Pericarp (mg/g)		Phenolic Content in Longan Seed (mg/g)	
	50% Ethanol Extract	70% Ethanol Extract	50% Ethanol Extract	70% Ethanol Extract
Gallic acid	0.07 ± 0.00 ^k^	0.08 ± 0.00 ^k^	0.47 ± 0.01 ^g^	0.49 ± 0.02 ^g^
Ethyl gallate	0.27 ± 0.01 ^h^	0.28 ± 0.03 ^h^	0.88 ± 0.02 ^f^	1.84 ± 0.06 ^d^
Ellagic acid	0.15 ± 0.00 ^j^	0.18 ± 0.00 ^i,j^	0.21 ± 0.01 ^i^	0.83 ± 0.00 ^f^
Corilagin	1.75 ± 0.05 ^e^	2.15 ± 0.08 ^c^	5.03 ± 0.03 ^b^	5.53 ± 0.05 ^a^

Values marked by different letters within a same column were significantly different (*P* < 0.05).

**Table 3:** IC_50_ values of DPPH radical scavenging activities of longan byproducts.

Sample	Phenolic Extracts (μg Extract/g)	Flavonoid Extracts (μg Extract/g)	Alkaloid Extracts (μg Extract/g)
Pericarp	1827.11 ± 16.64 ^a^	451.96 ± 3.62 ^c^	29.17 ± 0.10 ^e^
Seed	1007.89 ± 23.22 ^b^	427.66 ± 3.01 ^d^	22.90 ± 1.20 ^f^

Values marked by different letters within a same column were significantly different (*P* < 0.05).

**Table 4:** Nitrite scavenging activities of phenolic extracts from longan byproducts.

Sample	Reaction Time	Nitrite Scavenging Activity (%)				
		0.2 mL Extracts	0.4 mL Extracts	0.6 mL Extracts	0.8 mL Extracts	1.0 mL Extracts
Pericarp	10 min	47.85 ± 1.09 ^p^	55.69 ± 1.31 ^n^	67.28 ± 1.80 ^k^	73.03 ± 2.05 ^i^	80.46 ± 1.96 ^g,h^
	30 min	48.31 ± 0.53 ^p^	70.15 ± 1.41 ^j^	80.00 ± 0.53 ^g,h^	85.74 ± 1.39 ^e^	88.15 ± 0.22 ^d^
	60 min	60.62 ± 1.07 ^m^	79.18 ± 1.08 ^h^	87.90 ± 1.08 ^d^	90.97 ± 1.69 ^b,c^	94.26 ± 0.89 ^a^
Seed	10 min	42.70 ± 0.94 ^q^	59.67 ± 1.08 ^m^	70.37 ± 0.53 ^j^	78.55 ± 0.22 ^h^	83.13 ± 0.64 ^f^
	30 min	52.62 ± 0.22 ^o^	71.60 ± 1.35 ^i,j^	83.85 ± 0.78 ^e,f^	89.30 ± 1.28 ^c,d^	91.98 ± 1.93 ^b^
	60 min	64.30 ± 1.08 ^l^	82.00 ± 0.78 ^f,g^	90.74 ± 1.41 ^b,c^	94.75 ± 1.07 ^a^	95.99 ± 1.11 ^a^

Values within each sample marked by the different letters in the Table were significantly different (*P* < 0.05).

**Table 5:** Nitrite scavenging activities of flavonoid extracts from longan byproducts.

Sample	Reaction Time	Nitrite Scavenging Activity (%)				
		0.2 mL Extracts	0.4 mL Extracts	0.6 mL Extracts	0.8 mL Extracts	1.0 mL Extracts
Pericarp	10 min	50.27 ± 1.92 ^n^	58.22 ± 1.62 ^l^	68.37 ± 0.55 ^i,j^	73.37 ± 0.56 ^h^	75.97 ± 0.67 ^h^
	30 min	55.84 ± 2.08 ^m^	68.79 ± 2.91 ^i,j^	75.86 ± 0.29 ^h^	84.25 ± 0.62 ^f^	85.28 ± 1.28 ^d,e^
	60 min	60.73 ± 0.43 ^k^	79.46 ± 0.60 ^g^	86.20 ± 0.92 ^c,d^	91.14 ± 0.76 ^b^	92.04 ± 0.46 ^a,b^
Seed	10 min	42.76 ± 0.33 ^o^	55.69 ± 0.41 ^m^	60.81 ± 0.88 ^k^	66.55 ± 1.02 ^j^	69.69 ± 0.84 ^i^
	30 min	51.21 ± 0.20 ^n^	67.13 ± 2.25 ^j^	75.77 ± 1.10 ^h^	81.66 ± 0.17 ^f,g^	83.30 ± 1.21 ^e,f^
	60 min	60.80 ± 0.69 ^k^	76.48 ± 0.91 ^h^	83.89 ± 1.18 ^d,e,f^	88.07 ± 1.44 ^c^	93.64 ± 1.06 ^a^

Values within each sample marked by the different letters in the Table were significantly different (*P* < 0.05).

**Table 6:** Nitrite scavenging activities of alkaloid extracts from longan byproducts.

Sample	Reaction Time	Nitrite Scavenging Activity (%)				
		0.2 mL Extracts	0.4 mL Extracts	0.6 mL Extracts	0.8 mL Extracts	1.0 mL Extracts
Pericarp	10 min	38.14 ± 1.51 ^r^	51.83 ± 1.38 ^o^	59.78 ± 1.74 ^m^	62.53 ± 1.38^l^	68.53 ± 0.86 ^j^
	30 min	56.89 ± 2.92 ^n^	72.96 ± 0.45 ^h,i^	80.12 ± 1.12 ^g^	84.57 ± 1.12 ^e,f^	87.59 ± 1.35 ^d^
	60 min	70.26 ± 0.47 ^i,j^	85.85 ± 0.47 ^d,e^	91.96 ± 1.74 ^a,b,c^	92.26 ± 1.84 ^a,b^	93.08 ± 2.84 ^a^
Seed	10 min	29.18 ± 1.86 ^s^	41.98 ± 1.65 ^q^	48.22 ± 0.33 ^p^	56.35 ± 1.26 ^n^	61.58 ± 1.46 ^l,m^
	30 min	50.33 ± 1.35 ^o,p^	63.92 ± 1.42 ^l^	74.50 ± 1.07 ^h^	79.18 ± 1.02 ^g^	83.18 ± 0.77 ^f^
	60 min	62.58 ± 0.22 ^l^	79.47 ± 0.99 ^g^	85.93 ± 0.18 ^d,e^	89.53 ± 0.92 ^c^	90.45 ± 0.31 ^b,c^

Values within each sample marked by the different letters in the Table were significantly different (*P* < 0.05).
